# Prescription Sequence Symmetry Analysis (PSSA) to assess prescribing cascades: a step-by-step guide

**DOI:** 10.1186/s12874-023-02108-y

**Published:** 2024-01-11

**Authors:** Mandy R. S. Hendrix, Mustafa Yasar, Atiya K. Mohammad, Jacqueline G. Hugtenburg, Joost W. Vanhommerig, Ruveyda Gündoğan-Yilmaz, Patricia M. L. A. van den Bemt, Petra Denig, Fatma Karapinar-Carkıt

**Affiliations:** 1 Department of Clinical Pharmacy, OLVG Hospital, Amsterdam, The Netherlands; 2 Department of Clinical Pharmacy and Pharmacology, University of Groningen, University Medical Centre Groningen, Amsterdam, The Netherlands; 3 Department of Clinical Pharmacology, Amsterdam UMC, location VUMC, Amsterdam, The Netherlands; 4 Department of Research and Epidemiology, OLVG Hospital, Amsterdam, The Netherlands; 5 Department of Clinical Pharmacy & Toxicology, Maastricht University Medical Center +, Maastricht, the Netherlands; 6 CARIM School for Cardiovascular Diseases, Maastricht University, Maastricht, The Netherlands

**Keywords:** Sequence symmetry analysis, Prescribing cascade, Pharmacoepidemiology, Pharmacovigilance, Signal detection

## Abstract

Prescribing cascades occur when patients are prescribed medication to treat the adverse drug reaction of previously prescribed medication. Prescription sequence symmetry analysis (PSSA) can be used to assess the association between two medications in prescription or dispensing databases and thus the potential occurrence of prescribing cascades. In this article, a step-by-step guide is presented for conducting PSSA to assess prescribing cascades. We describe considerations for medication data collection and setting time periods for relevant parameters, including washout window, exposure window, continued exposure interval and blackout period. With two examples, we illustrate the impact of changes in these parameters on the strengths of associations observed. Given the impact seen, we recommend that researchers clearly specify and explain all considerations regarding medication included and time windows set when studying prescribing cascades with PSSA, and conduct subgroup and sensitivity analyses.

**Supplementary Information:**

The online version contains supplementary material available at 10.1186/s12874-023-02108-y.

## Background

A prescribing cascade has been defined as a misinterpretation of an adverse drug reaction (ADR) as a new medical condition, which is subsequently treated with another medication [1]. In some cases, treating or preventing an ADR with another medication is justified but when the ADR is not acknowledged as such, the resulting prescribing cascade is considered problematic. It is important to identify, manage or prevent such prescribing cascades because they can lead to polypharmacy, adverse outcomes and unnecessary healthcare costs [1]. In recent years, the number of studies addressing prescribing cascades is increasing [2].

To identify and quantify potential prescribing cascades prescription or administrative databases can be used. The *sequence symmetry analysis* (SSA) method has been used to assess the association between a medication and for example hospital diagnoses or aids (e.g. incontinence products). In the field of pharmacoepidemiology, the *prescription sequence symmetry analysis* (PSSA) method is used to assess the association between two medicines [2]. PSSA quantifies prescribing cascades with the sequence ratio (SR) as a risk estimate. The crude SR (cSR) is calculated by the number of patients who initiated the initial medication (i.e. index medication) first and the medication to treat the ADR (i.e. marker medication) second divided by the number of patients who initiated the marker medication first and the index medication second [3, 4]. This cSR is sensitive to prescribing trends over time, e.g., due to expired patents or a change in treatment guideline recommendations. To correct for prescribing trends, the null-effect SR (SRnull) is calculated. The null-effect SR takes the prescribing trends in the background population into account, by computing an expected SR based on the probability of the sequence of initiation of the marker medication after the index medication in the absence of any causal association [3]. By dividing the cSR by the SRnull, the adjusted SR (aSR) is calculated. If the aSR is more than 1.0, there is an increased probability that a prescribing cascade has occurred due to an ADR of the index medication [3].

PSSA is a useful method to identify the occurrence of potential prescribing cascades in clinical practice when the ADR is known or strongly hypothesized. PSSA is easy to implement, requiring prescription data that include a patient identifier and prescription dates [3]. Its conceptual framework but also statistical codes for calculating cSR, SRnull and aSR have been explained in previous papers [3, 5]. Previous studies, however, illustrate that different data collection methods, definitions and assumptions are used for PSSA [2, 3, 5–8]. So far, the considerations for medication data collection and setting of time periods for relevant parameters have not been described or discussed in detail. This is needed to support future studies in providing meaningful information for identifying prescribing cascades or evaluating of the effects of interventions to reverse or prevent prescribing cascades. Therefore, our aim is 1) to provide a step-by-step guide for executing PSSA to assess prescribing cascades, and 2) to show the impact of changing assumptions on aSRs using two examples: angiotensin-converting enzyme inhibitors (ACEi)-induced cough followed by antitussives, and cardiovascular medication-induced erectile dysfunction followed by phosphodiesterase inhibitors.

## Methods

**Fig. 1:**
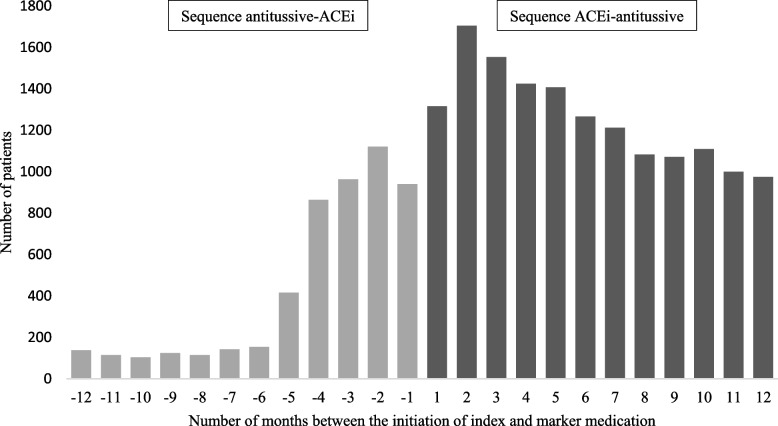
Prescribing pattern of the prescribing cascade ACEi – antitussive (*n* = 20,313 patients). The start of the ACEi (index medication) is set at month 0. The frequencies of the initial antitussive (marker medication) prescriptions are shown before (light grey bar charts) and after the start of the ACEi (dark grey bar charts). The asymmetrical prescribing pattern implies an association between the ACEi and the antitussive. Note that in the first months of the index and marker medication, there is an increase in both the light and dark grey bar charts. This phenomenon is often seen. This might be attributed to the initiation of one medication, leading to more healthcare visits and the initiation of other medication (i.e., medication initiation being temporally correlated with each other)

Previous papers have explained the PSSA method using a general pharmacoepidemiologic perspective [2–5]. We briefly describe the basic principles of PSSA to assess prescribing cascades using the ACEi-antitussives prescribing cascade as example. When there would be no causal relationship between the use of an ACEi and the need for an antitussive, the probability that a patient is prescribed an antitussive before or after the start of an ACEi is expected to be equal. This would result in a symmetrical or random prescribing pattern of the initiation of the marker medication around the initiation of the index medication. In contrast, when the ACEi leads to an increased probability of cough that in turn is treated with the antitussive, it would result in an asymmetrical prescribing pattern of initial prescriptions (Fig. 1).

To quantify the occurrence of prescribing cascades, one first determines the cSR as described in the introduction. Next, to adjust for prescribing trends over time, the null-effect SR (SRnull) is calculated. The SRnull is calculated with the following two formulas:

$$Pa=\frac{\sum_{m=1}^{\mu }[{I}_{m}* \left(\Sigma\;patients\;starting\;marker\;after\;start\;date\;index\right)] }{\sum_{m=1}^{\mu }[{I}_{m}* \left(\left(\Sigma\;patients\;starting\;marker\;prior\;to\;start\;date\;index\right)+\left(\Sigma\;patients\;starting\;marker\;after\;start\;date\;index\right)\right)]}$$

Here, Pa stands for the overall probability that the marker medication will be prescribed after the index medication when the prescription pattern of the background population is taken into consideration; *m* indicates the consecutive day of the index medication of the study and *µ* indicates the last day of the study period. *Im* is the number of persons receiving their first index medication on the specific day and the start date index is the first day an index medication is prescribed [3].

When the Pa is calculated, the null-effect SR can be calculated with the following formula:

$$Null-effect\;SR= \frac{Pa}{1-Pa}$$

By dividing the cSR by the SRnull, the aSR is calculated. For the detailed calculations of the cSR, SRnull, aSR with 95% confidence interval (95% CI), we refer to Electronic Supplementary Material (ESM) 1.

### Executing PSSA for prescribing cascades

#### Considerations for the data collection

Although collecting data to conduct a PSSA for a prescribing cascade may seem straightforward, several decisions have to be made (Table 1). First of all, sufficient history and a predefined follow-up period is needed. Since loss to follow-up could be non-random and the occurrence of true prescribing cascades may take some time, the effect of including patients with incomplete follow-up could bias the SR for the prescribing cascade. To define a patient as lost to follow-up, one should not limit this to only considering dispensings of the index or marker medication, but should also account for continuous enrollment when using claims datasets. To ensure continuous enrollment for patient eligibility, the presence of dispensings of any medication or other claims data during the period of interest can be used [7].

**Table 1:** Specifications and considerations relevant for the data collection

**1**	Specify at which medication class level the index medication will be collected. Is the ADR caused by a group of medication or an individual medication?
**2**	Specify at which medication class level the marker medication will be collected. Is the ADR treated by one individual medication or a medication group or with medication from different medication groups?
**3**	Specify which combination products will be included in the data collection for the index and/or marker medication.
**4**	Specify how it will be ensured that the patients have sufficient history and follow-up data. Which additional data will be used to ascertain continuous enrollment?
**5**	Consider characteristics of the ADR, such as the possible dose-relatedness of the ADR and when the ADR generally occurs to inform decisions on subgroup analysis and time windows.
**6**	Consider patient characteristics that might influence the development of the ADR (e.g. sex, age or comorbidities) to inform patient inclusion or subgroup analysis.
**7**	Consider co-medication that might influence the development of the ADR to inform patient inclusion or subgroup analysis.

##### Medication class level

Interest in quantifying a prescribing cascade may start with a particular case, for example, lisinopril followed by codeine which may be indicative of treating the ADR cough. The first consideration is whether the ADR cough is a group effect of the index medication or an ADR caused by this individual medication. As cough is indeed a known group side effect of ACEi [9], assessing the prescribing cascade at the class level as index medication makes more sense than at the individual substance level lisinopril. In contrast, for amiodarone-induced hypothyroidism including the group of all antiarrhythmics would be inappropriate since hypothyroidism is a specific ADR of amiodarone [10]. Similarly, for the selection of the marker medication, the medication class level needs to be considered. ACEi-induced cough can be treated with codeine but also with other antitussives [8]. Moreover, focussing on antitussives only could fail to show the complete spectrum of prescribing cascades related to ACEi-induced cough. Cough has also been treated with salbutamol, antihistamines or antibiotics [11–13], so to quantify the ACEi-induced cough prescribing cascade, a range of marker medication classes could be included. However, including too many marker medications that are not very specific for treating cough could result in lower SRs in the PSSA, as the chances to identify asymmetry in prescribing sequences of the index and marker medication will decrease. Sensitivity analyses regarding the class level could be used to test the robustness of the PSSA results.

##### Combination products

Next, inclusion of combination products of the index as well as the marker medication should be considered. For example, when assessing the prescribing cascade of calcium channel blocker (CCB)-induced edema treated with a diuretic, CCBs can be defined as the index medication. The initiation of diuretics and/or combination products of CCBs with diuretics can be defined as the marker medication. Inclusion or exclusion of combination products of CCBs with diuretics as marker medication can change the aSR. In contrast, the combination of CCBs with a beta-blocker can be defined as index medication, since beta-blockers are not likely to be prescribed for treating edema.

##### Dose dependence of ADR

Some ADRs can be related to the actual dose of medication. If an ADR is dose-related, a false negative result could be found in a database with a high prevalence of patients using low doses of the index medication. If a dose-relationship is expected, it could be relevant to perform subanalyses examining aSRs for low and high dose prescriptions.

##### Risk factors of ADR

Certain comorbidities -but also sex or age- may influence the development of the ADR and thus the likelihood of a prescribing cascade. For example, for the prescribing cascade ACEi – antitussives, having obstructive airway disease can induce or contribute to the outcome of dry cough [8]. Also, older patients are more likely to develop an ADR due to more comorbid conditions, polypharmacy and a higher sensitivity for medication effects [14]. Amiodarone-induced hypothyroidism is more frequently reported in women [10]. Therefore, the prescribing cascade could be more likely in women than in men. In such cases, it can be relevant to conduct subgroup analyses or select only the patients at risk of developing the ADR for identifying or quantifying the prescribing cascade.

##### Co-medication

**Fig. 2:**
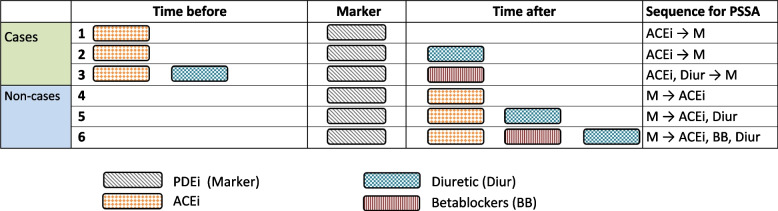
Schematic representation of the prescribing cascade cardiovascular medication induced erectile dysfunction treated with phosphodiesterase inhibitors. Cases = group of patients where the index medication(s) of interest is followed by the marker medication, non-cases = group of patients where the marker medication is followed by the index medication(s), ACEi = angiotensin converting enzyme inhibitor, BB = beta blocker, Diur = diuretic, M = marker medication, PDEi = Phosphodiesterase inhibitor

It is not uncommon that there is co-medication that could result in the same ADR and thus the same prescribing cascade. For example, the ADR erectile dysfunction has been documented for a variety of cardiovascular medication groups, such as ACEi, betablockers and diuretics. If the prescribing cascade of interest is solely ACEi-induced erectile dysfunction, one could include patients who only use ACEi (i.e., sole users, patients 1, 2 and 4 in Fig. 2). Patients who use other subgroups of cardiovascular medication could be excluded, as the sole effect of ACEi cannot be determined for these patients (Fig. 2, patients 3, 5 and 6, where the PSSA cannot differentiate between erectile dysfunction possibly caused by the ACEi versus the co-medication or due to a combination of both). Misclassifications can occur when co-medication is not adequately taken into account (Fig. 2, patients 2 and 3, where the sequence of phosphodiesterase (PDE) inhibitors followed by diuretics and betablockers respectively would be found when ACEi was disregarded). On the other hand, when the use of co-medication is unrelated to the index medication and its initiation is expected to be stable over the time period that is studied, it may not influence the asymmetry caused by the index medication of interest in the PSSA calculations.

Alternatively, multiple subgroup analyses could be performed to study the impact of comedication, e.g., for patients using one versus two or three and more classes of cardiovascular medication causing the same ADR. However, including too many different classes for the index medication may lead to more risk of bias, such as confounding by indication.

#### Parameters in PSSA analysis

There are a number of time windows that should be imposed **both** for the sequence index-marker as well as for the sequence marker-index when conducting PSSA to assess prescribing cascades. Relevant parameters that are often considered for PSSA include the *washout window* to identify incident users and the *exposure window* for assessing the associations. In addition, a *blackout period* and *continued exposure interval* (CEI), which have been defined in other pharmacoepidemiological studies, can be relevant when using PSSA for assessing prescribing cascades [15–17]. These parameters can best be imposed at medication episode level, for which a definition of medication discontinuation is needed. This could be equal to the period set for the washout window. Importantly, one should first establish continuous enrollment during the washout and the exposure window for patient eligibility (see also previous paragraph).

##### Washout window

**Fig. 3:**
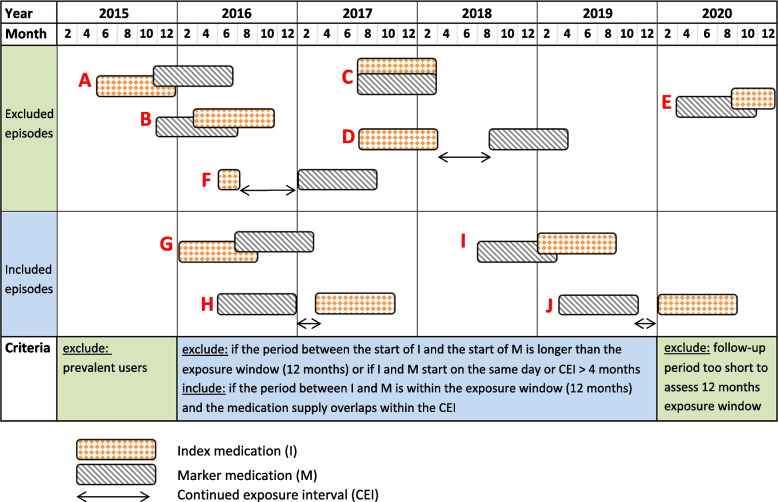
In- and exclusion criteria for PSSA assessment covering the period January 2015 to December 2020. The episodes shown are general examples of applying different time periods and do not relate to a specific combination of medications. Set parameters: 12 months washout window, 12 months exposure window, 0 days for the blackout period, and continued exposure interval (CEI) of 4 months. Example episodes A until F are all excluded based on the PSSA parameter settings. Example episodes G until J are included

The washout window (also called ‘waiting time period’ or ‘run-in period’) is the period that is imposed as a look-back period to ensure that the index or marker medication is indeed a first prescription (incidence) and exclude any prevalent users of the index or marker medication [4, 8, 18]. By selecting incident users, the initiation of the marker medication after an index medication is more likely to indicate the treatment for a new ADR than treatment for an ongoing medical condition [3]. Of note, dose-dependent ADRs may occur after dose increases in prevalent users and some ADRs only develop after continued exposure [19]. The washout window should depend on the maximum period that medication is dispensed in a country to make sure that any prevalent users are excluded. In the Netherlands, chronic medication is generally dispensed for 3 months but this can be extended to a maximum of 6 months. A washout window of 12 months could be considered suitable taking into account any medication supply a patient could have in stock, and reducing the chance of including prevalent users. In previous PSSA studies, the washout window was set at 6 or 12 months [2, 3]. Figure 3 shows examples of episodes that could be excluded because the criteria for the washout window are not met (examples A and B).

##### Exposure window

The exposure window (also called ‘exposure time window’ or ‘observation period’) is the defined follow-up period to capture the pairs of incident index and marker medication users and vice versa, so the maximum allowed time period between the start of the index and the start of the marker medication [20]. The exposure window should depend on the expected time onset of the ADR caused by the index medication [2, 18]. For example, Pouwels et al. used an exposure window of 4 weeks in their study of ACEi-induced urinary tract infections, as reduced urine output and reduced glomerular filtration rate (GFR) after ACEi initiation have been reported in relatively short-term studies ranging from 7 days to 8 weeks [21]. It should be noted that such a short period is likely to decrease the sample size and the precision of the SR calculations [22]. Also, a short exposure window can result in missing ADRs that take longer to occur, e.g., amiodarone can induce hypothyroidism even at 39 months after amiodarone initiation [23]. It should also be kept in mind that for some ADRs patients can have a delay in consulting a healthcare provider [10].

The downside of a longer exposure window is that the association between the index and the marker medication may become weaker and there is a higher risk of time-varying confounding [3, 4, 22]. Generally, a 12-month exposure window is used to reduce the impact of such confounding, which includes ageing, disease progression and other time-varying variables, such as change in diet and/or environment [20]. An exposure window of 12 months was found optimal for achieving acceptable sensitivity (61%; 95% CI 0.46–0.74) and high specificity (93%; 95% CI 0.87–0.96) for 165 tested index-marker pairs [22]. Choosing different time periods can provide insight regarding the onset time of an ADR. In Fig. 3, examples are shown of episodes that are included (example G, H, I, and J) and excluded (example D and E) when an exposure window of 12 months is used. Of note, in the final year of data collection, episodes of the marker or index medication are disregarded when the exposure window requirement can no longer be met (example E).

##### Continued exposure interval (CEI)

To define continued exposure to a drug, the continued exposure interval (CEI) (also called ‘maximum permissible length without medication supply’) needs to be defined. This is the gap between the expected end date of one prescription and the date of the next prescription. In many pharmacoepidemiological studies, this would refer to the exposure to one medication or medication class but it can also be applied when associations between pairs of medication are studied [15]. When studying prescribing cascades, the CEI is the maximum acceptable period without medication supply for the first medication [16]. This period is imposed to ensure patients are likely to be exposed to the first medication when initiating the other medication.

The CEI should be based on the common dispensing period of medication and the expected medication taking behaviour and could be similar to what is used in studies that measure adherence using dispensing data [16, 24]. For example, after the first prescription, medication prescriptions are generally repeated every 3 months in the Netherlands. Taking into account any supply the patient still possesses from previous dispensings (stockpile), a CEI of 4 to 6 months could be used. In Fig. 3, examples D and F show episodes that are excluded when a CEI of 4 months is imposed. It should be kept in mind that in most prescription and administrative databases the expected end date is theoretical and often determined based on the amount dispensed and the dose instruction when available. For medication that is prescribed without a clear dose instruction (e.g., use as needed), determining the expected end date can be difficult. Alternatively, one can set a longer period after the start date of each prescription, similarly to the definition of a discontinuation as well as the washout period, to exclude cases where impact of the initial medication is no longer expected. When imposing the CEI it should be kept in mind that some medication can still result in ADRs after discontinuation.

##### Blackout period

The blackout period (also called ‘time-lag period’ and ‘lag time’) is the period immediately after the initiation of the first medication in which events, i.e. the start of the other medication, will not be taken into account. Such a period is relevant to ensure sufficient time for the development of the ADR induced by the index medication and subsequent treatment with the marker medication [17]. Singh et al. studied the prescribing cascade of calcium channel blocker (CCB)-induced edema treated with diuretics [17]. They imposed a blackout period of 7 days because the probability of developing edema within 7 days after initiating CCBs is low. Other studies mentioned that patients need to be excluded if they have their first prescription of the index and marker medication on the exact same date, as the sequence of the index and marker medication cannot be extracted (see Fig. 3, example C) [7, 8]. Formally, this is not defined as blackout period but is important to address as exclusion criterion. Although an ADR may occur immediately, it seems impossible that a prescribing cascade occurs on the day of the first prescription.

## Results

To illustrate the impact of including co-medication and using different assumptions on the calculated aSRs, we used a dataset obtained from Ncontrol. Ncontrol holds data of dispensed prescriptions of more than 600 affiliated Dutch community pharmacies [25]. Data were retrieved from January 1st 2015 until December 31st 2020. The prescribing cascade cardiovascular medication-induced erectile dysfunction was used to show the effect of including co-medication for three subgroups of cardiovascular medication. The prescribing cascade ACEi-antitussives was used to show the effect of different assumptions regarding the parameters needed for PSSA (i.e., washout window, exposure window, CEI and blackout period). The syntax for use in IBM SPSS Statistics version 27 (IBM Corporation, Armonk, New York, U.S.) is included in ESM 2 and an additional file is necessary to make the syntax work (ESM 3). The fictional sample dataset of ACEi-induced cough is included in ESM 4.

### Impact of co-medication on PSSA

In Table 2 the PSSA calculations are shown for sole users of respectively an ACEi, a beta-blocker or a high ceiling diuretic for the prescribing cascade erectile dysfunction. The aSR found for these sole users are somewhat lower than when any user of respectively an ACEi, a beta-blocker or a high ceiling diuretic is included in the PSSA calculations, with overlapping CIs. For example, including any ACEi user resulted in an aSR of 1.91 (95% CI: 1.84–1.99), while including sole ACEi users resulted in an aSR of 1.74 (95% CI: 1.62–1.86). When patients using two subgroups are included in the PSSA calculation the aSR increases to 2.18 (95% CI: 2.10–2.27) compared to sole users. For patients using a combination of three subgroups of cardiovascular medication, the increase is even higher with an aSR of 2.81 (95% CI: 2.69–2.93). These subgroup analyses confirm that this prescribing cascade is not specific for a particular medication group.

**Table 2:** Calculations of sequence ratios incorporating co-medication for the prescribing cascade of three cardiovascular medication groups-induced erectile dysfunction and patients using multiple groups

	**Study population**	**Patients using index before marker**	**Patients using marker before index**	**cSR**	**SRnull**	**aSR (95% CI)**
*Any ACEi users*^*a*^	3,188	2,152	1,036	2.08	1.09	1.91 (1.84-1.99)
*Sole ACEi users*^*b*^	1,170	767	403	1.90	1.09	1.74 (1.62-1.86)
*Any beta blocker users *	3,035	1,937	1,098	1.76	1.06	1.66 (1.58-1.73)
*Sole beta blocker users*	1,228	739	489	1.51	1.05	1.44 (1.33-1.56)
*Any high ceiling diuretic users *	688	405	283	1.43	1.04	1.38 (1.23-1.53)
*Sole high ceiling diuretic users *	342	200	142	1.41	1.04	1.36 (1.14-1.57)
*Combination: use of two subgroups*^*c*^	2,419	1,695	724	2.34	1.07	2.18 (2.10-2.27)
*Combination: use of three or more subgroups *	1,544	1,167	377	3.10	1.10	2.81 (2.69-2.93)

*ACEi* angiotensin converting enzyme inhibitor, *cSR* crude sequence ratio, *SRnull* null-effect sequence ratio, *aSR* adjusted sequence ratio, *95% CI* 95% confidence interval

^a^Any users are patients that use other medication that can cause the same adverse drug reaction

^b^Sole users are patients that use NO other medication that can cause the same adverse drug reaction

^c^Combination users are patients that use multiple medication that can cause the same adverse drug reaction

Note: Combination users used any combination of subgroups (i.e., ACEi, beta blocker, high ceiling diuretics, low ceiling diuretics, calcium channel blockers, angiotensin II blockers, aldosterone antagonists and statins)

### Impact of changing assumptions on PSSA

To illustrate the effect of different assumptions for the previously discussed parameters used in PSSA on the aSR, calculations have been made on the ACEi – antitussive medication prescribing cascade (Table 3). In the primary analysis, the washout window and the exposure window were 12 months, the CEI was 4 months, and the blackout period was 7 days. The aSR of the primary analysis was 2.59 (95% CI: 2.56–2.62). In the second analysis, a washout window of 6 months was used instead of 12 months. This included more patients compared to the primary analysis and resulted in a higher aSR 2.73 (95% CI: 2.70–2.76). The higher aSR suggests a stronger association. In the third analysis, a 6-months exposure window was used instead of 12 months. This analysis included less patients as the follow-up period was shorter and resulted in a lower aSR of 1.80 (95% CI: 1.77–1.84). This may be the result of missing some of the more delayed prescribing cascades. In the fourth analysis, a 6-months CEI was used instead of 4 months. The aSR was lower 2.07 (95% CI: 2.04–2.10) than the first analysis, indicating that by choosing a longer period for the CEI, the association will be weaker as this analysis includes more patients initiating the marker medication for a non-related indication. In the final analysis, no blackout period was considered instead of a 7-days blackout period. This resulted in a similar aSR 2.52 (95% CI: 2.49–2.55) compared to the primary analysis. The chance that patients initiate antitussive medication within 7 days after initiating ACEi therapy (and vice versa) is relatively low, so the effect of implementing a short blackout period in this analysis was minimal.

**Table 3:** Calculations of sequence ratios for the prescribing cascade ACEi-antitussives using different assumptions

	**Study population**	**Patients using index before marker**	**Patients using marker before index**	**cSR**	**SRnull**	**aSR (95% CI)**
*Primary analysis*^*a*^	20,313	15,121	5,192	2.91	1.13	2.59 (2.56-2.62)
*Washout window 6 months *	29,809	22,357	7,452	3.00	1.10	2.73 (2.70-2.76)
*Exposure window 6 months *	16,382	10,699	5,683	1.88	1.04	1.80 (1.77-1.83)
*CEI 6 months*	22,256	15,469	6,787	2.28	1.10	2.07 (2.04-2.10)
*No blackout period*^*b*^	20,959	15,478	5,481	2.82	1.12	2.52 (2.49-2.55)

*CEI* continued exposure interval, *cSR* crude sequence ratio, *SRnull* null-effect sequence ratio, *aSR* adjusted sequence ratio, *95% CI* 95% confidence interval

^a^The assumptions of the parameters used in the primary analysis were: washout window 12 months, exposure window 12 months, CEI 4 months and blackout period 7 days

^b^Patients are excluded if they have their first prescription of the index and marker medication on the same date, as the sequence of the index and marker medication cannot be determined

## Discussion and conclusions

PSSA has several strong points. First, the method has been validated in a study including 19 medications showed high specificity (93%) and moderate sensitivity (61%) for identifying ADRs [22]. This indicates that PSSA is likely to identify many albeit not all prescribing cascades. In an overview of this method, Lai et al. stated that PSSA can capture prescribing cascades even when the ADR is rare [3]. Second, PSSA is easy to implement using dispensing or prescription databases as little information is required for the basic calculations, that is, a patient identifier and the prescription dates for the index and marker medication. Third, PSSA is robust to patient confounders that are stable over time, such as sex and genetic factors, because PSSA is based on within-subject comparison [3]. Several pitfalls of PSSA have been summarized which are typical of observational research, including time-varying confounding, protopathic bias or confounding by indication (i.e., the indication of the index medication leads to prescription of the marker medication) [3]. Over the years, some approaches have been proposed to improve the application of the PSSA method, such as conducting sensitivity analyses and including positive controls (with known prescribing cascades) or negative controls (with unrelated marker medication) to test the robustness of the results [2, 26, 27].

We add to this by providing a detailed description of considerations that are relevant for data collection and analysis when applying PSSA to assess prescribing cascades. Several considerations are common for the conduct of pharmacoepidemiological studies using prescription or dispensing databases, including setting a washout period to identify incident users and defining medication discontinuation to identify the end of a medication episode. When studying prescribing cascades, additional information about the ADR is needed for setting the time periods for the exposure window and blackout period. Of note, dose-dependent ADRs may occur after dose increases in prevalent users, requiring additional subgroup analyses. In addition, we have illustrated how comedication can influence the PSSA results when quantifying prescribing cascades. It might be relevant to study the prescribing cascade at a high medication class level or to stratify for combined medication use. In the examples provided in this study, we showed that changing the washout window, exposure window, CEI and blackout period can impact the strength of the associations observed. Although the overall direction of the association was not changed in our examples this could be different for other prescribing cascades, for example, when the aSR is close to one. This illustrates that conducting sensitivity analyses regarding all these parameters is relevant. Of note, when conducting sensitivity analyses for the exposure window, it is important to adjust the time periods for the null-effect SR as well. There are no rules for selecting the medication classes or setting the optimal time windows. We recommend that researchers clearly specify and explain all considerations regarding the index and marker medication included and the time windows set when studying prescribing cascades with PSSA. With this, information about prescribing cascades can be generated that is needed to address prescribing cascades in the future.

### Supplementary Information

**Additional file 1.** 

**Additional file 2.** 

**Additional file 3.** 

**Additional file 4.** 

## Data Availability

The datasets generated during and analyzed during the current study are available from the corresponding author on reasonable request.
