# Supplementary material for: Prescription Sequence Symmetry Analysis (PSSA) to assess prescribing cascades: a step-by-step guide
Source: BMC Med Res Methodol. 2024 Jan 11;24:8. doi: 10.1186/s12874-023-02108-y (PMC10782776; doi:10.1186/s12874-023-02108-y)
Supplement: Supplementary file 1 — Additional file 1. [file 12874_2023_2108_MOESM1_ESM.pdf]

Supplementary data 1: Calculations in PSSA

The crude sequence ratio (cSR) is calculated by dividing the number of patients that start the index medication first followed by the marker medication with the number of patients who start the marker medication first followed by the index medication with the following formula (Lai EC-C, et al. Sequence symmetry analysis in pharmacovigilance and pharmacoepidemiologic studies. Eur J Epidemiol. 2017;32(7):567-82):

$$cSR = \frac{number\ of\ patients\ starting\ an\ index\ medication \rightarrow marker\ medication}{number\ of\ patients\ starting\ a\ marker\ medication \rightarrow index\ medication}$$

For example, if there are 746 incident users that start with the index medication first and 423 patients that start with the marker medication first, the cSR would be 746/423 = 1.76.

The calculation of the cSR is sensitive to changes in prescribing trends over time, e.g. changes in treatment guidelines, expired patents or distribution problems. For example, if reimbursement for medication A increased during the study period while for medication B it remained stable, this could result in a non-random excess of prescribing of medication A. Therefore, to adjust for prescribing trends a null-effect sequence ratio is calculated with the following formula:

$$Pa = \frac{\sum_{m=1}^{\mu}[I_m * \ (\sum_{n=m+1}^{m+d} M_n)]}{\sum_{m=1}^{\mu}[I_m * ((\sum_{n=m-d}^{m-1} M_n) + (\sum_{n=m+1}^{m+d} M_n))]}$$

Here, Pa stands for the overall probability that the marker medication will be prescribed after the index medication when the prescription pattern of the background population is taken into consideration.

μ indicates the last day of the study period

m indicates the consecutive day of the index medication of the study

Im is the number of persons receiving their first index medication on the specific day

d indicates the exposure window in days

n indicates the consecutive days of the study period

Mn is the number of persons receiving their first marker medication on the specific day. The number of patients with a first marker medication is always within the set exposure window from *Im*.

To understand the formula better, the formula can be simplified into:

$$Pa = \frac{\sum_{m=1}^{\mu}[I_m * \ (\Sigma\, patients\ starting\ marker\ after\ start\ date\ index)]}{\sum_{m=1}^{\mu}[I_m * \ ((\Sigma\, patients\ starting\ marker\ prior\ to\ start\ date\ index) + (\Sigma\, patients\ starting\ marker\ after\ start\ date\ index))]}$$

To explain how this formula can be applied, the fictional data below are used (table 1). For this example, the study period is seven days and the exposure window is three days. In the first example, only data from day 1 until day 7 is used. For each day in the study period the number of patients starting with their first index medication and the number of patients starting with their first marker medication is presented.

Table 1. Fictional data on the first prescription of the index medication and the marker medication (incident users) each day for 14 days.

Day	*Number of patients starting with index medication*	*Number of patients starting with marker medication*
**1**	2	0
**2**	1	3
**3**	3	2
**4**	3	2
**5**	1	3
**6**	2	4
**7**	0	0
**8**	8	8
**9**	2	2
**10**	6	1
**11**	7	3
**12**	3	0
**13**	4	3
**14**	7	4

The probability that the marker medication will be prescribed after the index medication for day 4 (framed in table 1) can be calculated using the following information:

μ = **7** days (study period)

m = day **4** for the index medication

Im = **3** patients started the index medication on day 4

d = **3** days

n = from day **5 (*m+1)*** for the consecutive days of the study period

$\sum_{n=m+1}^{m+d} M_n$ = 7; **3 patients (day 5) + 4 patients (day 6) + 0 patients (day 7)** receive the marker medication after the start date of the index

$\sum_{n=m-1}^{m-d} M_n$ = 5; **0 patients (day 1) + 3 patients (day 2) + 2 patients (day 3)** receive the marker medication prior to start date index

This information can be applied in the formula:

$$Pa\ day\ 4 = \frac{\sum_{4=1}^{7}[3 * \ (\sum_{4+1}^{4+3} M_n)]}{\sum_{4=1}^{7}[3 * ((\sum_{4-3}^{4-1} M_n) + (\sum_{4+1}^{4+3} M_n))]}$$

=

$$Pa\ day\ 4 = \frac{\sum_{4=1}^{7}[3 * \ (3 + 4 + 0)]}{\sum_{4=1}^{7}[3 * ((0 + 3 + 2) + \ (3 + 4 + 0))]}$$

=

$$Pa\ day\ 4 = \frac{\sum_{4=1}^{7}[21]}{\sum_{4=1}^{7}[36]}$$

For the calculation of Pa for all days, all the seven days of the study period need to be calculated (the example data on day four are in bold):

$$Pa = \frac{[14 + 7 + 27 + \mathbf{21} + 4 + 0 + 0]}{[14 + 7 + 36 + \mathbf{36} + 11 + 14 + 0]} = 0.62$$

When the Pa is calculated, the null-effect sequence ratio can be calculated with the following formula:

$$Null - effect\ sequence\ ratio = \frac{Pa}{1-Pa} = \frac{0.62}{1-0.62} = 1.62$$

Finally, the adjusted sequence ratio (aSR) is calculated. If the cSR was 1.76, the aSR would be:

$$aSR = \frac{cSR}{Null-effect\ sequence\ ratio} = \frac{1.76}{1.62} = 1.09$$

To determine the standard error (SE) and confidence interval (CI) of the calculated cSR and/or aSRs, the following formulas are used:

$$SE = \sqrt{(\frac{1}{group\ 1}) + (\frac{1}{group2})}$$

$$Lower\ limit\ CI = aSR - (1.96 * SE)$$

$$Upper\ limit\ CI = aSR + (1.96 * SE)$$

Group 1= the number of people with the index medication followed by the marker medication

Group 2= the number of people with the marker medication followed by the index medication

Following the same example as before, the 95% CI is calculated as follows:

$$SE = \sqrt{(\frac{1}{746}) + (\frac{1}{423})} = 0.06$$

$$Lower\ limit\ CI = 1.09 - (1.96 * 0.06) = 0.97$$

$$Upper\ limit\ CI = 1.09 + (1.96 * 0.06) = 1.21$$

So for the study period of day 1 – day 7, the aSR is 1.09 (95% CI [0.97 - 1.21])

Different null-effect sequence ratio

To illustrate that the null-effect sequence ratio can differ depending on the studied period, the null-effect sequence ratio is calculated with a second example with a study period of day 8 – day 14 (see table 1).

The calculations for the Pa and for the null-effect sequence ratio for day 8 – day 14 are as follows:

$$Pa = \frac{[24 + 8 + 36 + 49 + 21 + 16 + 0]}{[24 + 24 + 96 + 70 + 39 + 32 + 42]} = 0.47$$

$$Null - effect\ sequence\ ratio = \frac{Pa}{1-Pa} = \frac{0.47}{1-0.47} = 0.89$$

This shows that the null-effect sequence ratio can both be >1.0 and <1.0 depending on the studied data.

If the cSR for the second example (study period of day 8 – day 14) was also 1.76, the aSR would be:

$$aSR = \frac{cSR}{Null-effect\ sequence\ ratio} = \frac{1.76}{0.89} = 1.98$$

The calculation of the 95% CI is as follows:

$$Lower\ limit\ CI = 1.98 - (1.96 * 0.06) = 1.86$$

$$Upper\ limit\ CI = 1.98 + (1.96 * 0.06) = 2.10$$

So for the study period day 8 – day 14, the aSR is 1.98 (95% CI [1.86 - 2.10]).
