# Supplementary material for: Prescription Sequence Symmetry Analysis (PSSA) to assess prescribing cascades: a step-by-step guide
Source: BMC Med Res Methodol. 2024 Jan 11;24:8. doi: 10.1186/s12874-023-02108-y (PMC10782776; doi:10.1186/s12874-023-02108-y)
Supplement: Supplementary file 2 — Additional file 2. [file 12874_2023_2108_MOESM2_ESM.pdf]

Supplemental data 2: syntax for PSSA calculations.

```
* Encoding: UTF-8.
*STEP  1. DEFINING THE PATH WHERE DATASETS ARE SAVED.
*Encoding : UTF-8.
*Define the path (folder on hard drive) where you will save all datasets for a prescribing cascade and where other temporary files can be stored.
*In this syntax the dataset “ACE-antitussives” is used.
*In addition, a dataset is needed with all dates of the study period to calculate the aSR (see step 14 where further explanation is provided for the dataset “ESM 3.sav”).
*During the execution of this syntax multiple datasets need to be saved to check for multiple episodes of index/marker medication and to perform the aSR calculations.
*Therefore, it is essential to define the path were all files are saved. Copy paste the right path (url hard drive) between the apostrophes. Note that before the last apostroph a \ sign needs to be present.
DEFINE !path() 'your own url hard drive\' !ENDDEFINE.

*Open the raw dataset and copy paste the SPSS filename between the apostrophes. End the filename with .sav.
GET FILE=!path+'ACE-antitussives.sav'.
DATASET NAME DataSet1 WINDOW=FRONT.
DATASET ACTIVATE DataSet1.


*STEP 2. DETERMINING THE NUMBER OF UNIQUE PATIENTS
*identify duplicate cases to calculate how many primary cases (i.e. unique patients) there are in the whole dataset, as one patient can have multiple rows.
*Note that the result here should be 126 patients.
SORT CASES BY PatientID(A).
MATCH FILES
  /FILE=*
  /BY PatientID
  /FIRST=PrimaryFirst
  /LAST=PrimaryLast.
DO IF (PrimaryFirst).
COMPUTE MatchSequence=1-PrimaryLast.
ELSE.
COMPUTE MatchSequence=MatchSequence+1.
END IF.
LEAVE MatchSequence.
FORMATS MatchSequence (f7).
COMPUTE InDupGrp=MatchSequence>0.
SORT CASES InDupGrp(D).
MATCH FILES
  /FILE=*
  /DROP=PrimaryFirst InDupGrp MatchSequence.
VARIABLE LABELS PrimaryLast 'Indicator of each last matching case as Primary'.
VALUE LABELS PrimaryLast 0 'Duplicate Case' 1 'Primary Case'.
VARIABLE LEVEL PrimaryLast (ORDINAL).
FREQUENCIES VARIABLES=PrimaryLast.
EXECUTE.

*STEP 3. SELECTING ADULT PATIENTS: inclusion criteria in this study
*COMPUTE the age based on the year of birth and select adults.
COMPUTE age=(2020 -Year_birth).
execute.
VARIABLE LABELS age 'The age of patients in the year 2020'.
SELECT IF age>=18.
EXECUTE.

*identify duplicate cases to calculate how many primary cases (i.e. unique adult patients) are left.
*Note that the result here should be 124 unique adult patients.
```

```
SORT CASES BY PatientID(A).
MATCH FILES
 /FILE=*
 /BY PatientID
 /FIRST=PrimaryFirst
 /LAST=PrimaryLast1.
DO IF (PrimaryFirst).
COMPUTE MatchSequence1=1-PrimaryLast1.
ELSE.
COMPUTE MatchSequence1=MatchSequence1+1.
END IF.
LEAVE MatchSequence1.
FORMATS MatchSequence1 (f7).
COMPUTE InDupGrp=MatchSequence1>0.
SORT CASES InDupGrp(D).
MATCH FILES
 /FILE=*
 /DROP=PrimaryFirst InDupGrp MatchSequence1.
VARIABLE LABELS PrimaryLast1 'Indicator of each last matching case as Primary'.
VALUE LABELS PrimaryLast1 0 'Duplicate Case' 1 'Primary Case'.
VARIABLE LEVEL PrimaryLast1 (ORDINAL).
FREQUENCIES VARIABLES=PrimaryLast1.
EXECUTE.

*STEP 4. CHECKING FOR MULTIPLE EPISODES.
* Theoretically, a patient can have four episodes in our study period as an incident user (data collection in this syntax was from 2015 until 2020).
* The first year (2015) is excluded as it cannot be determined whether the patient is an incident user. But, for example, if a patient starts an index medication at the beginning of 2015 (and does not receive this medication anymore for one year) an episode could start when a new index medication is initiated again.
* IF the same patient receives the index medication in March 2016 (the washout window of 12 months is met) this episode needs to be checked for inclusion criteria. IF the marker medication starts for example in August 2016, the CEI criterion (in this syntax four months) is not met and this episode will also be excluded.
* IF both medication again are discontinued (for 12 months) and in November 2017 a new episode starts, this episode also needs to be checked for the inclusion criteria.
* The same implies for the year 2018 and 2019.
* The check for multiple episodes is repeated four times (for 2016, 2017, 2018, 2019). Medication starting in 2020 is excluded as the exposure window requirement (in this syntax 12 months) is not met.
* Of note: The FIRST episode of index and marker medication (and vice versa) that meets all inclusion criteria is included for the aSR calculation.
* In the following commands multiple episodes are identified by identifying whether both the index and marker medication were discontinued (e.g. not dispensed for a period>12 monts).
SORT CASES by patientid (a) order_dispensing(a).
COMPUTE previous_dispensing=0.
VARIABLE LABELS previous_dispensing 'The date of the previous dispensing'.
IF patientid=lag(patientid) previous_dispensing=lag(dispensing_date).
FORMATS previous_dispensing (edate10).
EXECUTE.

*Calculate the time between the current and each previous dispensing for a patient.
*NOTE: this serves as a check for the washout window later.
SORT CASES by patientid (a) order_dispensing(a).
IF patientid=lag(patientid) timediff_dispensing=(dispensing_date-previous_dispensing)/(60*60*24).
VARIABLE LABELS timediff_dispensing 'The time difference in days between the actual dispensing date and the date of the previous dispensing'.
EXECUTE.

*Label difference in dispensing time IF >12 months (i.e. the washout window) to evaluate IF patients have discontinued the medication previously. Discontinued is defined in this syntax as a timediff_dispensing>12 months (to adjust for leap years, 365.25 days is used to defined the washout window).
```

IF timediff_dispensing>365.25 episodes=1.
VARIABLE LABELS episodes 'A period of discontinuation of minimally one year in the medication history of a patient (labelled with 1)'.
EXECUTE.

*The order of discontinations has to be numbered to know which one came first. For this reason, discontinued episodes are sorted by descending order.
SORT CASES by Patientid (a) episodes (d) dispensing_date (a).
IF patientid=lag(patientid) episodes=lag(episodes)+1.
EXECUTE.

*This file should be saved as the main dataset that can be used to identify all further episodes (see, the later steps 9-11) that meet the inclusion criteria.
SAVE OUTFILE = !path + 'motherfile.sav'.
DATASET NAME episodes1.
EXECUTE.

*STEP 5. CALCULATING THE TIME-INTERVAL
*It is necessary to know what the interval in time is between the start of the first and second medication to check whether episodes fulfil the criterion for exposure window (i.e. the follow-up period, 12 months in this syntax)
*(see step 6 where patients fulfilling the exposure window criterion are eventually identified).
*To organize all the rows of data for each patient, sort them based on their ID and the order of all the dispensings they had.
*The very first time an index medication is dispensed to each patient needs to be identified with a new variable 'first_medication'. The dataset is sorted on showing the index medication first for every patient. Then, to identify a dIFferent patient, the ~ sign (=not equal to) is used. Lag means ‘not the same as’.
SORT CASES by PatientID(a) indexORmarker(a) Order_dispensing(a).
IF PatientID~=lag(PatientID) and IndexORmarker=1 first_medication=1.
VARIABLE LABELS first_medication 'The first index or marker medication that has been prescribed'.
execute.

*The command above cannot identify the very first row per patient, that's why the command below is used. The sign $ is used to continue numbering.
COMPUTE casenum=$casenum.
VARIABLE LABELS casenum 'Each row within the dataset is numbered chronologically'.
IF $CASENUM=1 and IndexORmarker=1 first_medication=1.
EXECUTE.

* The very first time a marker medication is dispensed to each patient over the whole study period needs to be identified also within the variable 'first_medication'. Note that the dataset needs to be sorted with the marker medication first.
SORT CASES by PatientID(a) IndexORmarker(d) Order_dispensing(a).
IF PatientID~=lag(PatientID) and IndexORmarker=2 first_medication=1.
COMPUTE casenum=$casenum.
IF $CASENUM=1 and IndexORmarker=2 first_medication=1.
EXECUTE.

*The exposure window of the episode is calculated with the dispensing dates of the first index and first marker of each patient.
*identify the dispensing date for the first marker per patient (new variable 'date_marker').
IF IndexORmarker=2 and first_medication=1 date_marker= Dispensing_date.
FORMATS date_marker (edate10).
VARIABLE LABELS date_marker 'The date of the first marker for each patient'.
EXECUTE.

*Copy the dispensing date for the first marker for each row of a patient to perform future calculations.
SORT CASES by PatientID(a) date_marker(d).
IF PatientID=lag(PatientID) date_marker=lag(date_marker).
EXECUTE.

```
*Identify the dispensing date for the first index per patient (new variable ‘date_index’).
IF IndexORmarker=1 and first_medication=1 date_index= Dispensing_date.
FORMATS date_index (edate10).
VARIABLE LABELS date_index 'The date of the first index for each patient'.
EXECUTE.

*Copy the dispensing date for the first index for each row of a patient to perform future calculations.
SORT CASES by PatientID(a) date_index(d).
IF PatientID=lag(PatientID) date_index=lag(date_index).
EXECUTE.

*Sorts patients over time in correct order.
SORT CASES by PatientID(a) dispensing_date (a).
EXECUTE.

*Calculate the exposure window for the first episode that occurs.
* NOTE: time will be calculated in seconds, so multiply to calculate days.
* Sequence index medication followed by marker medication results in a positive number of days.
* Sequence marker medication followed by index medication results in a negative number of days.
COMPUTE exposure_window=(date_marker - date_index)/(60*60*24).
VARIABLE LABELS exposure_window 'The time in days between the first marker and the first index'.
EXECUTE.


* STEP 6. SELECTING PATIENTS WITH THE SET EXPOSURE WINDOW AND BLACKOUT PERIOD
*Episodes fulfilling the criteria for the exposure window need to be selected (12 months in this syntax). This will exclude
patients that were lost to follow up due to death, moving away, etc. These patients are identified as having no
dispensing of any medication.
*First, the exposure window criterion is calculated. For the sequence index medication followed by the marker
medication: the exposure window >=0 is selected and 12 months is added to the first dispensing date of the index
medication.
IF exposure_window>=0 followup=date_index+(365.25*24*60*60).
FORMATS followup (edate10).
VARIABLE LABELS followup 'The minimal follow-up date for an episode'.
EXECUTE.

*The exposure window for the sequence marker medication followed by an index medication is calculated: the exposure
window<0 is selected and 12 months is added to the first dispensing date of the marker medication.
IF exposure_window<0 followup=date_marker+(365.25*24*60*60).
EXECUTE.

*Check IF the exposure window (i.e. follow-up of 12 months) is completed for each patient.
IF PatientID =lag(PatientID) and dispensing_date>=followup completefollowup=1.
EXECUTE.

*Copy this value to all rows of a patient, so this patient is identified as a patient with a complete exposure window.
SORT CASES by PatientID(a) completefollowup(d).
IF PatientID=lag(PatientID) completefollowup=lag(completefollowup).
VARIABLE LABELS completefollowup 'Label to identify patients that meet the inclusion of follow up (1= minimal 1 year
follow up, sysmis=<1 year follow up)'.
EXECUTE.

*check the number of patients with a complete follow-up: here 1721.
FREQUENCIES VARIABLES=completefollowup
 /STATISTICS=RANGE MINIMUM MAXIMUM MODE
 /ORDER=ANALYSIS.

*Select patients who have completed the exposure window.
*Note: patients with an incomplete period are removed from the dataset.
SORT CASES by patientid (a) Dispensing_date (a).
```

```
SELECT IF completefollowup=1.
EXECUTE.

*Recheck whether right number of patients with a complete follow-up are selected: here again 1721.
FREQUENCIES VARIABLES=completefollowup
 /STATISTICS=RANGE MINIMUM MAXIMUM MODE
 /ORDER=ANALYSIS.

*COMPUTE a new variable to label the rows within the selected exposure windows (in this syntax 12 months).
*The blackout period (in this syntax 7 days) is also incorporated in this command.
COMPUTE exposure_window_1yr=0.
IF exposure_window>-365.25 and exposure_window<-7 exposure_window_1yr=1.
IF exposure_window<365.25  and exposure_window>7 exposure_window_1yr=1.
VARIABLE LABELS exposure_window_1yr 'Label to identify if episodes meet inclusion criteria exposure window (12 months follow-up) and the black-out period (7 days)'.
EXECUTE.

*Check the number of patients.
FREQUENCIES VARIABLES=exposure_window_1yr
 /STATISTICS=RANGE MINIMUM MAXIMUM MODE
 /ORDER=ANALYSIS.

*Select the patients that meet the final inclusion criteria for exposure window and blackout period.
SELECT IF exposure_window_1yr=1.
EXECUTE.

*Recheck whether the same number of patients are found after selection of patients. Here 1196.
FREQUENCIES VARIABLES=exposure_window_1yr
 /STATISTICS=RANGE MINIMUM MAXIMUM MODE
 /ORDER=ANALYSIS.

* STEP 7. INCORPORATING THE CONTINUED EXPOSURE INTERVAL (CEI).
*Identify the rows that are between the first index and marker and vice versa, this way the focus is only on the episode of prescribing cascade.
if indexORmarker=1 and dispensing_date<=date_marker episodes_rows=1.
if indexORmarker=2 and dispensing_date<=date_index episodes_rows=1.
EXECUTE.

*Select the rows of the episode only.
select if first_medication=1 or episodes_rows=1.
EXECUTE.

*Identify what the sequence of the episode is Sequence12=first index then marker. Sequence 21 is marker first then index.
if order_dispensing=1 and IndexOrmarker=1 sequence=12.
if order_dispensing=1 and IndexOrmarker=2 sequence=21.
EXECUTE.

*Copy this value for each row of a patient (as this patient is included).
SORT CASES by PatientID(a) sequence(d).
IF PatientID =lag(PatientID) sequence=lag(sequence).
EXECUTE.

*Calculate the days between the start date of the first time a marker medication is prescribed and the end date of each row containing an index medication.
IF sequence=12 and indexORmarker=1 continuedexposure_im=(date_marker- Enddate)/(60*60*24).
VARIABLE LABELS continuedexposure_im 'The continued exposure window of episodes for the sequence index-->marker in days'.
EXECUTE.
```

```
*Generate a new variable to label each row that has the selected CEI (in this syntax 4 months).
IF continuedexposure_im < 365.25/3 CEI_im=1.
VARIABLE LABELS CEI_im 'Label to identify whether episodes meet the continued exposure interval criteria of 4 months'.
EXECUTE.

*Calculate the days between the start date of the first index medication and the end date of each row containing the marker medication.
IF sequence=21 and IndexORmarker=2 continuedexposure_mi=(date_index- Enddate)/(60*60*24).
VARIABLE LABELS continuedexposure_mi 'The continued exposure window of episodes for the sequence marker-->index in days'.
EXECUTE.

* Generate a new variable to label each row that has the selected CEI (in this syntax 4 months).
IF continuedexposure_mi < 365.25/3 CEI_mi=1.
VARIABLE LABELS CEI_mi 'Label to identify whether episodes meet the continued exposure intervenal criteria of 4 months'.
EXECUTE.

*Copy this value for each row of a patient (as this patient is included).
SORT CASES by PatientID(a) CEI_im(d).
IF PatientID =lag(PatientID) CEI_im=lag(CEI_im).
EXECUTE.

*Copy this value for each row of a patient (as this patient is included).
SORT CASES by PatientID(a) CEI_mi(d).
IF PatientID =lag(PatientID) CEI_mi=lag(CEI_mi).
EXECUTE.

*Compute a “selection” variable to label all patients with a first index and marker medication with the selected CEI (<= 4 months).
COMPUTE selection=0.
IF first_medication=1 and CEI_im=1 selection=1.
IF first_medication=1 and CEI_mi=1 selection=1.
VARIABLE LABELS selection 'Label to select the correct rows of patients with a first index or marker medication fulfulling the continued exposure interval'.
EXECUTE.

*Check the number of patients.
FREQUENCIES VARIABLES=selection
 /STATISTICS=RANGE MINIMUM MAXIMUM MODE
 /ORDER=ANALYSIS.

*Select the patients.
*NOTE: after this command only two rows for each patient will be left as these are the index and marker medication that fulfil the previous inclusion criteria.
SELECT IF selection=1.
EXECUTE.

*Recheck whether the same number of patients are found after selection of patients. Here 118.
FREQUENCIES VARIABLES=selection
 /STATISTICS=RANGE MINIMUM MAXIMUM MODE
 /ORDER=ANALYSIS.

*STEP 8. INCORPORATING THE WASHOUT WINDOW
*An index medication or marker medication starting in the first year of data collection needs to be excluded as it cannot be determined whether these prescriptions are for incident or prevalent users (washout window).
*Compute a new variable 'keep'.
COMPUTE keep=1.
VARIABLE LABELS keep 'Selection of patients who fullfill the washout criterion'.
```

```
EXECUTE.

*All dispensings of the first year (in this syntax 2015) should be excluded, defined as 'keep=0'.
IF Dispensing_date<date.dmy(1,1,2016) keep=0.
EXECUTE.

*Copy this keep=0 for each row of a patient to exclude this episode (as for at least one of the medication it cannot be determined IF the medication is for an incident user and not prevalence).
SORT CASES by patientid(a) keep(a).
IF patientid=lag(patientid) and lag(keep)=0 keep=0.
EXECUTE.

*Select the patients with keep=1. This is the first dataset with patients who met all inclusion criteria (adults, exposure window, blackout period, continued exposure interval, washout window).
SELECT IF keep=1.
EXECUTE.

*Save the file with all the identified first episodes and episodes1 should contain 108 rows.
FREQUENCIES VARIABLES=PatientID
  /FORMAT=NOTABLE
  /ORDER=ANALYSIS.
SAVE OUTFILE= !path+'episodes1.sav'.


*STEP 9. CHECKING FOR SECOND EPISODES
*Go to the dataset "motherfile" that sorts the patients dispensings (starting from the second episode, see step 4 for the explanation on multiple episodes).
GET FILE = !path + 'motherfile.sav'.
DATASET NAME episodes2.
DATASET CLOSE episodes1.

*A new variable is computed to number the order dispensing starting from the second episode in one patient.
IF episodes=1 numbering=1.
VARIABLE LABELS numbering 'The new numbering taking a discontinuation into account'.
COMPUTE diff_numbering=(Order_dispensing-numbering).
VARIABLE LABELS diff_numbering 'The difference in numbering when considering new episodes after a discontinuation'.
recode diff_numbering (SYSMIS=999).
EXECUTE.

*Copy the value for the difference for each row of the patient.
*Patients can have multiple discontinuation episodes, but in the command below the most recent discontinuation will be taken into account.  Later, other episodes will also be checked (see step 10 and 11).
SORT CASES by PatientID(a) diff_numbering(a).
IF PatientID=lag(PatientID) diff_numbering=lag(diff_numbering).
EXECUTE.

*Third, COMPUTE the new order of dispensing for the second episode.
COMPUTE order_dispensing2=(order_dispensing-diff_numbering).
VARIABLE LABELS order_dispensing2 'The order of dispensing of medication for each patient, where 1 is the first'.
EXECUTE.

*Select the order dispensing from the second episode.
*Rename the variable order_dispensing2 into the original variable name order_dispensing. This is necessary for aggregating all the data into one dataset at the end of the selection of episodes within one patient.
SORT CASES by patientid(a) order_dispensing2(a).
SELECT IF order_dispensing2>0.
EXECUTE.
DELETE VARIABLES order_dispensing.
RENAME VARIABLES (order_dispensing2=order_dispensing).
```

EXECUTE.

*Now all parameters for PSSA need to be checked again, for the explanation per command, see step 5, 6, 7 (note that step 8 is not necessary as the washout window already has been selected based on the calculations in step 4 for all episodes).
*The explanations on the repeated steps 5-7 here will be brief.
*Rerun step 5 for the time-interval of the second episode.
SORT CASES by PatientID(a) indexORmarker(a) Order_dispensing(a).
IF PatientID~=lag(PatientID) and IndexORmarker=1 first_medication=1.
VARIABLE LABELS first_medication 'The first index or marker medication that has been prescribed'.
execute.

*The command above cannot identify the very first row per patient, that's why the command below is used. The sign $ is used to continue numbering.
COMPUTE casenum=$casenum.
VARIABLE LABELS casenum 'Each row within the dataset is numbered chronologically'.
IF $CASENUM=1 and IndexORmarker=1 first_medication=1.
EXECUTE.

* The very first time a marker medication is dispensed to each patient over the whole study period needs to be identified also within the variable 'first_medication'. Note that the dataset needs to be sorted with the marker medication first.
SORT CASES by PatientID(a) IndexORmarker(d) Order_dispensing(a).
IF PatientID~=lag(PatientID) and IndexORmarker=2 first_medication=1.
COMPUTE casenum=$casenum.
IF $CASENUM=1 and IndexORmarker=2 first_medication=1.
EXECUTE.

*The exposure window of the episode is calculated with the dispensing dates of the first index and first marker of each patient.
*identify the dispensing date for the first marker per patient (new variable 'date_marker').
IF IndexORmarker=2 and first_medication=1 date_marker= Dispensing_date.
FORMATS date_marker (edate10).
VARIABLE LABELS date_marker 'The date of the first marker for each patient'.
EXECUTE.

*Copy the dispensing date for the first marker for each row of a patient to perform future calculations.
SORT CASES by PatientID(a) date_marker(d).
IF PatientID=lag(PatientID) date_marker=lag(date_marker).
EXECUTE.

*Identify the dispensing date for the first index per patient (new variable ‘date_index’).
IF IndexORmarker=1 and first_medication=1 date_index= Dispensing_date.
FORMATS date_index (edate10).
VARIABLE LABELS date_index 'The date of the first index for each patient'.
EXECUTE.

*Copy the dispensing date for the first index for each row of a patient to perform future calculations.
SORT CASES by PatientID(a) date_index(d).
IF PatientID=lag(PatientID) date_index=lag(date_index).
EXECUTE.

*Sorts patients over time in correct order.
SORT CASES by PatientID(a) dispensing_date (a).
EXECUTE.

*Calculate the exposure window for the first episode that occurs.
* NOTE: time will be calculated in seconds, so multiply to calculate days.
* Sequence index medication followed by marker medication results in a positive number of days.
* Sequence marker medication followed by index medication results in a negative number of days.
COMPUTE exposure_window=(date_marker - date_index)/(60*60*24).

```
VARIABLE LABELS exposure_window 'The time in days between the first marker and the first index'.
EXECUTE.


* STEP 6. SELECTING PATIENTS WITH THE SET EXPOSURE WINDOW AND BLACKOUT PERIOD
*Episodes fulfilling the criteria for the exposure window need to be selected (12 months in this syntax). This will exclude patients that were lost to follow up due to death, moving away, etc. These patients are identified as having no dispensing of any medication.
*First, the exposure window criterion is calculated. For the sequence index medication followed by the marker medication: the exposure window >=0 is selected and 12 months is added to the first dispensing date of the index medication.
IF exposure_window>=0 followup=date_index+(365.25*24*60*60).
FORMATS followup (edate10).
VARIABLE LABELS followup 'The minimal follow-up date for an episode'.
EXECUTE.

*The exposure window for the sequence marker medication followed by an index medication is calculated: the exposure window<0 is selected and 12 months is added to the first dispensing date of the marker medication.
IF exposure_window<0 followup=date_marker+(365.25*24*60*60).
EXECUTE.

*Check IF the exposure window (i.e. follow-up of 12 months) is completed for each patient.
IF PatientID =lag(PatientID) and dispensing_date>=followup completefollowup=1.
EXECUTE.

*Copy this value to all rows of a patient, so this patient is identified as a patient with a complete exposure window.
SORT CASES by PatientID(a) completefollowup(d).
IF PatientID=lag(PatientID) completefollowup=lag(completefollowup).
VARIABLE LABELS completefollowup 'Label to identify patients that meet the inclusion of follow up (1= minimal 1 year follow up, sysmis=<1 year follow up)'.
EXECUTE.

*check the number of patients with a complete follow-up: here 1721.
FREQUENCIES VARIABLES=completefollowup
 /STATISTICS=RANGE MINIMUM MAXIMUM MODE
 /ORDER=ANALYSIS.

*Select patients who have completed the exposure window.
*Note: patients with an incomplete period are removed from the dataset.
SORT CASES by patientid (a) Dispensing_date (a).
SELECT IF completefollowup=1.
EXECUTE.

*Recheck whether right number of patients with a complete follow-up are selected: here again 1721.
FREQUENCIES VARIABLES=completefollowup
 /STATISTICS=RANGE MINIMUM MAXIMUM MODE
 /ORDER=ANALYSIS.

*COMPUTE a new variable to label the rows within the selected exposure windows (in this syntax 12 months).
*The blackout period (in this syntax 7 days) is also incorporated in this command.
COMPUTE exposure_window_1yr=0.
IF exposure_window>-365.25 and exposure_window<-7 exposure_window_1yr=1.
IF exposure_window<365.25  and exposure_window>7 exposure_window_1yr=1.
VARIABLE LABELS exposure_window_1yr 'Label to identify if episodes meet inclusion criteria exposure window (12 months follow-up) and the black-out period (7 days)'.
EXECUTE.

*Check the number of patients.
FREQUENCIES VARIABLES=exposure_window_1yr
 /STATISTICS=RANGE MINIMUM MAXIMUM MODE
```

```
 /ORDER=ANALYSIS.

*Select the patients that meet the final inclusion criteria for exposure window and blackout period.
SELECT IF exposure_window_1yr=1.
EXECUTE.

*Recheck whether the same number of patients are found after selection of patients. Here 1196.
FREQUENCIES VARIABLES=exposure_window_1yr
 /STATISTICS=RANGE MINIMUM MAXIMUM MODE
 /ORDER=ANALYSIS.

* STEP 7. INCORPORATING THE CONTINUED EXPOSURE INTERVAL (CEI).
*Identify the rows that are between the first index and marker and vice versa, this way the focus is only on the episode
of prescribing cascade.
if indexORmarker=1 and dispensing_date<=date_marker episodes_rows=1.
if indexORmarker=2 and dispensing_date<=date_index episodes_rows=1.
EXECUTE.

*Select the rows of the episode only.
select if first_medication=1 or episodes_rows=1.
EXECUTE.

*Identify what the sequence of the episode is Sequence12=first index then marker. Sequence 21 is marker first then
index.
if order_dispensing=1 and IndexOrmarker=1 sequence=12.
if order_dispensing=1 and IndexOrmarker=2 sequence=21.
EXECUTE.

*Copy this value for each row of a patient (as this patient is included).
SORT CASES by PatientID(a) sequence(d).
IF PatientID =lag(PatientID) sequence=lag(sequence).
EXECUTE.

*Calculate the days between the start date of the first time a marker medication is prescribed and the end date of each
row containing an index medication.
IF sequence=12 and indexORmarker=1 continuedexposure_im=(date_marker- Enddate)/(60*60*24).
VARIABLE LABELS continuedexposure_im 'The continued exposure window of episodes for the sequence
index-->marker in days'.
EXECUTE.

*Generate a new variable to label each row that has the selected CEI (in this syntax 4 months).
IF continuedexposure_im < 365.25/3 CEI_im=1.
VARIABLE LABELS CEI_im 'Label to identify whether episodes meet the continued exposure interval criteria of 4
months'.
EXECUTE.

*Calculate the days between the start date of the first index medication and the end date of each row containing the
marker medication.
IF sequence=21 and IndexORmarker=2 continuedexposure_mi=(date_index- Enddate)/(60*60*24).
VARIABLE LABELS continuedexposure_mi 'The continued exposure window of episodes for the sequence
marker-->index in days'.
EXECUTE.

* Generate a new variable to label each row that has the selected CEI (in this syntax 4 months).
IF continuedexposure_mi < 365.25/3 CEI_mi=1.
VARIABLE LABELS CEI_mi 'Label to identify whether episodes meet the continued exposure intervenal criteria of 4
months'.
EXECUTE.

*Copy this value for each row of a patient (as this patient is included).
```

```
SORT CASES by PatientID(a) CEI_im(d).
IF PatientID =lag(PatientID) CEI_im=lag(CEI_im).
EXECUTE.

*Copy this value for each row of a patient (as this patient is included).
SORT CASES by PatientID(a) CEI_mi(d).
IF PatientID =lag(PatientID) CEI_mi=lag(CEI_mi).
EXECUTE.

*Compute a “selection” variable to label all patients with a first index and marker medication with the selected CEI (<= 4 months).
COMPUTE selection=0.
IF first_medication=1 and CEI_im=1 selection=1.
IF first_medication=1 and CEI_mi=1 selection=1.
VARIABLE LABELS selection 'Label to select the correct rows of patients with a first index or marker medication fulfulling the continued exposure interval'.
EXECUTE.

*Check the number of patients.
FREQUENCIES VARIABLES=selection
 /STATISTICS=RANGE MINIMUM MAXIMUM MODE
 /ORDER=ANALYSIS.

*Select the patients.
*NOTE: after this command only two rows for each patient will be left as these are the index and marker medication that fulfil the previous inclusion criteria.
SELECT IF selection=1.
EXECUTE.

*Save the file with all the identified first episodes and episodes2 should contain 22 rows.
FREQUENCIES VARIABLES=PatientID
  /FORMAT=NOTABLE
  /ORDER=ANALYSIS.
SAVE OUTFILE= !path+'episodes2.sav'.


 *STEP 10. SELECTING THE THIRD EPISODE
* The same steps as in step 9 are implemented for the third episode.
GET FILE = !path+'motherfile.sav'.
DATASET NAME episodes3.
DATASET CLOSE episodes2.

IF timediff_dispensing>365.25 and episodes=2 numbering=1.
VARIABLE LABELS numbering 'The new numbering taking discontination into account'.
COMPUTE diff_numbering=(Order_dispensing-numbering).
VARIABLE LABELS diff_numbering 'The difference in numbering when considering new episodes after discontinuation'.
EXECUTE.
recode diff_numbering (SYSMIS=999).
EXECUTE.

SORT CASES by PatientID(a) diff_numbering(a).
IF PatientID=lag(PatientID) diff_numbering=lag(diff_numbering).
EXECUTE.

COMPUTE order_dispensing2=(order_dispensing-diff_numbering).
VARIABLE LABELS order_dispensing2 'The order of dispensing of medication for each patient, where 1 is the first'.
EXECUTE.
SORT CASES by patientid(a) order_dispensing2(a).
EXECUTE.
```

```
SELECT IF order_dispensing2>0.
EXECUTE.

DELETE VARIABLES order_dispensing.
RENAME VARIABLES (order_dispensing2=order_dispensing).
EXECUTE.

SORT CASES by PatientID(a) indexORmarker(a) Order_dispensing(a).
IF PatientID~=lag(PatientID) and IndexORmarker=1 first_medication=1.
VARIABLE LABELS first_medication 'The first index or marker medication that has been prescribed'.
execute.

COMPUTE casenum=$casenum.
VARIABLE LABELS casenum 'Each row within the dataset is numbered chronologically'.
IF $CASENUM=1  and IndexORmarker=1  first_medication=1.
EXECUTE.

SORT CASES by PatientID(a) IndexORmarker(d) Order_dispensing(a).
IF PatientID~=lag(PatientID) and IndexORmarker=2 first_medication=1.
COMPUTE casenum=$casenum.
IF $CASENUM=1  and IndexORmarker=2  first_medication=1.
EXECUTE.

IF IndexORmarker=2 and first_medication=1 date_marker= Dispensing_date.
FORMATS date_marker (edate10).
VARIABLE LABELS date_marker 'The date of the first marker for each patient'.
EXECUTE.

SORT CASES by PatientID(a) date_marker(d).
IF PatientID=lag(PatientID) date_marker=lag(date_marker).
EXECUTE.

IF IndexORmarker=1 and first_medication=1 date_index= Dispensing_date.
FORMATS date_index (edate10).
VARIABLE LABELS date_index 'The date of the first index for each patient'.
EXECUTE.

SORT CASES by PatientID(a) date_index(d).
IF PatientID=lag(PatientID) date_index=lag(date_index).
EXECUTE.

SORT CASES by PatientID(a) dispensing_date (a).
EXECUTE.

COMPUTE exposure_window=(date_marker - date_index)/(60*60*24).
VARIABLE LABELS exposure_window 'The time in days between the first marker and the first index'.
EXECUTE.

IF exposure_window>=0 followup=date_index+(365.25*24*60*60).
FORMATS followup (edate10).
VARIABLE LABELS followup 'The minimal follow-up date for an episode'.
EXECUTE.

IF exposure_window<0 followup=date_marker+(365.25*24*60*60).
EXECUTE.

IF PatientID =lag(PatientID) and dispensing_date>=followup completefollowup=1.
EXECUTE.

SORT CASES by PatientID(a) completefollowup(d).
```

```
IF PatientID=lag(PatientID) completefollowup=lag(completefollowup).
VARIABLE LABELS completefollowup 'Label to identify patients that meet the inclusion of follow up (1= minimal 1 year follow up, sysmis=<1 year follow up)'.
EXECUTE.

FREQUENCIES VARIABLES=completefollowup
 /STATISTICS=RANGE MINIMUM MAXIMUM MODE
 /ORDER=ANALYSIS.

SORT CASES by patientid (a) Dispensing_date (a).
SELECT IF completefollowup=1.
EXECUTE.

FREQUENCIES VARIABLES=completefollowup
 /STATISTICS=RANGE MINIMUM MAXIMUM MODE
 /ORDER=ANALYSIS.

COMPUTE exposure_window_1yr=0.
IF exposure_window>-365.25 and exposure_window<-7 exposure_window_1yr=1.
IF exposure_window<365.25  and exposure_window>7 exposure_window_1yr=1.
VARIABLE LABELS exposure_window_1yr 'Label to identify if episodes meet inclusion criteria exposure window (12 months follow-up) and the black-out period (7 days)'.
EXECUTE.

FREQUENCIES VARIABLES=exposure_window_1yr
 /STATISTICS=RANGE MINIMUM MAXIMUM MODE
 /ORDER=ANALYSIS.

SELECT IF exposure_window_1yr=1.
EXECUTE.

FREQUENCIES VARIABLES=exposure_window_1yr
 /STATISTICS=RANGE MINIMUM MAXIMUM MODE
 /ORDER=ANALYSIS.

if indexORmarker=1 and dispensing_date<=date_marker episodes_rows=1.
if indexORmarker=2 and dispensing_date<=date_index episodes_rows=1.
EXECUTE.

select if first_medication=1 or episodes_rows=1.
EXECUTE.

if order_dispensing=1 and IndexOrmarker=1 sequence=12.
if order_dispensing=1 and IndexOrmarker=2 sequence=21.
EXECUTE.

SORT CASES by PatientID(a) sequence(d).
IF PatientID =lag(PatientID) sequence=lag(sequence).
EXECUTE.

IF sequence=12 and indexORmarker=1 continuedexposure_im=(date_marker- Enddate)/(60*60*24).
VARIABLE LABELS continuedexposure_im 'The continued exposure window of episodes for the sequence index-->marker in days'.
EXECUTE.

IF continuedexposure_im < 365.25/3 CEI_im=1.
VARIABLE LABELS CEI_im 'Label to identify whether episodes meet the continued exposure interval criteria of 4 months'.
EXECUTE.
```

```
IF sequence=21 and IndexORmarker=2 continuedexposure_mi=(date_index- Enddate)/(60*60*24).
VARIABLE LABELS continuedexposure_mi 'The continued exposure window of episodes for the sequence
marker-->index in days'.
EXECUTE.

IF continuedexposure_mi < 365.25/3 CEI_mi=1.
VARIABLE LABELS CEI_mi 'Label to identify whether episodes meet the continued exposure intervenal criteria of 4
months'.
EXECUTE.

SORT CASES by PatientID(a) CEI_im(d).
IF PatientID =lag(PatientID) CEI_im=lag(CEI_im).
EXECUTE.

SORT CASES by PatientID(a) CEI_mi(d).
IF PatientID =lag(PatientID) CEI_mi=lag(CEI_mi).
EXECUTE.

COMPUTE selection=0.
IF first_medication=1 and CEI_im=1 selection=1.
IF first_medication=1 and CEI_mi=1 selection=1.
VARIABLE LABELS selection 'Label to select the correct rows of patients with a first index or marker medication
fulfulling the continued exposure interval'.
EXECUTE.

FREQUENCIES VARIABLES=selection
 /STATISTICS=RANGE MINIMUM MAXIMUM MODE
 /ORDER=ANALYSIS.

SELECT IF selection=1.
EXECUTE.


*episodes3 should contain 16 rows.
FREQUENCIES VARIABLES=PatientID
  /FORMAT=NOTABLE
  /ORDER=ANALYSIS.
SAVE OUTFILE= !path+'episodes3.sav'.


*STEP 11: SELECTING THE FOURTH  EPISODE
* The same steps as in step 9 are implemented for the fourth episode.
GET FILE = !path+'motherfile.sav'.
DATASET NAME episodes4.
DATASET CLOSE episodes3.

IF timediff_dispensing>365.25 and episodes=3 numbering=1.
VARIABLE LABELS numbering 'The new numbering taking discontinuation into account'.
COMPUTE diff_numbering=(Order_dispensing-numbering).
VARIABLE LABELS diff_numbering 'The difference in numbering when considering new episodes after discontinuation'.
EXECUTE.
recode diff_numbering (SYSMIS=999).
EXECUTE.

SORT CASES by PatientID(a) diff_numbering(a).
IF PatientID=lag(PatientID) diff_numbering=lag(diff_numbering).
EXECUTE.

COMPUTE order_dispensing2=(order_dispensing-diff_numbering).
VARIABLE LABELS order_dispensing2 'The order of dispensing of medication for each patient, where 1 is the first'.
```

```
EXECUTE.
SORT CASES by patientid(a) order_dispensing2(a).
EXECUTE.

SELECT IF order_dispensing2>0.
EXECUTE.

DELETE VARIABLES order_dispensing.
RENAME VARIABLES (order_dispensing2=order_dispensing).
EXECUTE.

SORT CASES by PatientID(a) indexORmarker(a) Order_dispensing(a).
IF PatientID~=lag(PatientID) and IndexORmarker=1 first_medication=1.
VARIABLE LABELS first_medication 'The first index or marker medication that has been prescribed'.
execute.

COMPUTE casenum=$casenum.
VARIABLE LABELS casenum 'Each row within the dataset is numbered chronologically'.
IF $CASENUM=1  and IndexORmarker=1  first_medication=1.
EXECUTE.

SORT CASES by PatientID(a) IndexORmarker(d) Order_dispensing(a).
IF PatientID~=lag(PatientID) and IndexORmarker=2 first_medication=1.
COMPUTE casenum=$casenum.
IF $CASENUM=1  and IndexORmarker=2  first_medication=1.
EXECUTE.

IF IndexORmarker=2 and first_medication=1 date_marker= Dispensing_date.
FORMATS date_marker (edate10).
VARIABLE LABELS date_marker 'The date of the first marker for each patient'.
EXECUTE.

SORT CASES by PatientID(a) date_marker(d).
IF PatientID=lag(PatientID) date_marker=lag(date_marker).
EXECUTE.

IF IndexORmarker=1 and first_medication=1 date_index= Dispensing_date.
FORMATS date_index (edate10).
VARIABLE LABELS date_index 'The date of the first index for each patient'.
EXECUTE.

SORT CASES by PatientID(a) date_index(d).
IF PatientID=lag(PatientID) date_index=lag(date_index).
EXECUTE.

SORT CASES by PatientID(a) dispensing_date (a).
EXECUTE.

COMPUTE exposure_window=(date_marker - date_index)/(60*60*24).
VARIABLE LABELS exposure_window 'The time in days between the first marker and the first index'.
EXECUTE.

IF exposure_window>=0 followup=date_index+(365.25*24*60*60).
FORMATS followup (edate10).
VARIABLE LABELS followup 'The minimal follow-up date for an episode'.
EXECUTE.

IF exposure_window<0 followup=date_marker+(365.25*24*60*60).
EXECUTE.
```

```
IF PatientID =lag(PatientID) and dispensing_date>=followup completefollowup=1.
EXECUTE.

SORT CASES by PatientID(a) completefollowup(d).
IF PatientID=lag(PatientID) completefollowup=lag(completefollowup).
VARIABLE LABELS completefollowup 'Label to identify patients that meet the inclusion of follow up (1= minimal 1 year follow up, sysmis=<1 year follow up)'.
EXECUTE.

FREQUENCIES VARIABLES=completefollowup
 /STATISTICS=RANGE MINIMUM MAXIMUM MODE
 /ORDER=ANALYSIS.

SORT CASES by patientid (a) Dispensing_date (a).
SELECT IF completefollowup=1.
EXECUTE.

FREQUENCIES VARIABLES=completefollowup
 /STATISTICS=RANGE MINIMUM MAXIMUM MODE
 /ORDER=ANALYSIS.

COMPUTE exposure_window_1yr=0.
IF exposure_window>-365.25 and exposure_window<-7 exposure_window_1yr=1.
IF exposure_window<365.25  and exposure_window>7 exposure_window_1yr=1.
VARIABLE LABELS exposure_window_1yr 'Label to identify if episodes meet inclusion criteria exposure window (12 months follow-up) and the black-out period (7 days)'.
EXECUTE.

FREQUENCIES VARIABLES=exposure_window_1yr
 /STATISTICS=RANGE MINIMUM MAXIMUM MODE
 /ORDER=ANALYSIS.

SELECT IF exposure_window_1yr=1.
EXECUTE.

FREQUENCIES VARIABLES=exposure_window_1yr
 /STATISTICS=RANGE MINIMUM MAXIMUM MODE
 /ORDER=ANALYSIS.

if indexORmarker=1 and dispensing_date<=date_marker episodes_rows=1.
if indexORmarker=2 and dispensing_date<=date_index episodes_rows=1.
EXECUTE.

select if first_medication=1 or episodes_rows=1.
EXECUTE.

if order_dispensing=1 and IndexOrmarker=1 sequence=12.
if order_dispensing=1 and IndexOrmarker=2 sequence=21.
EXECUTE.

SORT CASES by PatientID(a) sequence(d).
IF PatientID =lag(PatientID) sequence=lag(sequence).
EXECUTE.

IF sequence=12 and indexORmarker=1 continuedexposure_im=(date_marker- Enddate)/(60*60*24).
VARIABLE LABELS continuedexposure_im 'The continued exposure window of episodes for the sequence index-->marker in days'.
EXECUTE.

IF continuedexposure_im < 365.25/3 CEI_im=1.
```

```
VARIABLE LABELS CEI_im 'Label to identify whether episodes meet the continued exposure interval criteria of 4
months'.
EXECUTE.

IF sequence=21 and IndexORmarker=2 continuedexposure_mi=(date_index- Enddate)/(60*60*24).
VARIABLE LABELS continuedexposure_mi 'The continued exposure window of episodes for the sequence
marker-->index in days'.
EXECUTE.

IF continuedexposure_mi < 365.25/3 CEI_mi=1.
VARIABLE LABELS CEI_mi 'Label to identify whether episodes meet the continued exposure intervenal criteria of 4
months'.
EXECUTE.

SORT CASES by PatientID(a) CEI_im(d).
IF PatientID =lag(PatientID) CEI_im=lag(CEI_im).
EXECUTE.

SORT CASES by PatientID(a) CEI_mi(d).
IF PatientID =lag(PatientID) CEI_mi=lag(CEI_mi).
EXECUTE.

COMPUTE selection=0.
IF first_medication=1 and CEI_im=1 selection=1.
IF first_medication=1 and CEI_mi=1 selection=1.
VARIABLE LABELS selection 'Label to select the correct rows of patients with a first index or marker medication
fulfulling the continued exposure interval'.
EXECUTE.

FREQUENCIES VARIABLES=selection
 /STATISTICS=RANGE MINIMUM MAXIMUM MODE
 /ORDER=ANALYSIS.

SELECT IF selection=1.
EXECUTE.

*episodes4 should contain 6 rows.
FREQUENCIES VARIABLES=PatientID
  /FORMAT=NOTABLE
  /ORDER=ANALYSIS.
SAVE OUTFILE = !path+'episodes4.sav'.


 *STEP 12. MERGING ALL EPISODES
*Add all the cases from the dIFferent episodes datasets into a new dataset and save it under the name "all_episodes".
DATASET ACTIVATE episodes4.
ADD FILES /FILE=*
  /FILE= !path+'episodes3.sav'.
EXECUTE.
ADD FILES /FILE=*
  /FILE=!path+'episodes2.sav'.
EXECUTE.
ADD FILES /FILE=*
  /FILE=!path+'episodes1.sav'.
EXECUTE.
SAVE OUTFILE= !path+'all_episodes.sav'.

*Check if the episodes were added correctly. In this case there should be (6+16+22+108=) 152 rows.
FREQUENCIES VARIABLES=PatientID
  /FORMAT=NOTABLE
```

```
  /ORDER=ANALYSIS.


*STEP 13 SELECTING THE FIRST SEQUENCE OCCURRING THAT MEETS ALL INCLUSION CRITERIA.
*At this point, possible multiple episodes of the index and marker medication pair are identified within one patient.
*Only, the FIRST occurring index/marker (or marker/index) pair is finally included, as this is the first episode that determines in what sequence the medication was dispensed to the patient.
*The first step is to identify the first index given to each patient over the study period. Give the value of 1 to the first index in the new variable 'first_medication'.
*NOTE: sorting is by dispensing_date here, not order_dispensing. This is because the first episode in time needs to be identified.
COMPUTE first_cascade=0.
SORT CASES by PatientID (a) IndexORmarker(a) Dispensing_date (a).
IF PatientID~=lag(PatientID) and IndexORmarker=1 first_cascade=1.
COMPUTE casenum1=$casenum.
IF $CASENUM=1  and IndexORmarker=1  first_cascade=1.
EXECUTE.

*Also, identify the first marker.
SORT CASES by PatientID(a) IndexORmarker(d) Dispensing_date (a).
IF PatientID~=lag(PatientID) and IndexORmarker=2 first_cascade=1.
COMPUTE casenum1=$casenum.
IF $CASENUM=1  and IndexORmarker=2  first_cascade=1.
EXECUTE.

*Select the index and marker (this is the first episode that is included as a prescribing cascade) with the value 1. This corresponds with the first occurring pair of index and marker medication (and vice versa).
fre first_cascade.
SELECT IF first_cascade=1.
EXECUTE.

*After only selecting the first cascade, 138 rows should remain.
FREQUENCIES VARIABLES=PatientID
  /FORMAT=NOTABLE
  /ORDER=ANALYSIS.

*Clean up the dataset by removing variables that are not necessary anymore.
DELETE VARIABLES
previous_dispensing
timediff_dispensing
episodes
numbering
diff_numbering
first_medication
casenum
followup
completefollowup
exposure_window_1yr
continuedexposure_im
CEI_im
selection
continuedexposure_mi
CEI_mi
episodes
keep
first_cascade
casenum1.
EXECUTE.

*Save this file under another name to save the patients with their first cascade occuring. For now the rows of patients
```

```
are not yet aggregated.
SAVE OUTFILE=!path +'all_cascades.sav'.

*IMPORTANT: sort patients based on their dispensing date, to make sure the order of dispensing is correct when the rows are rearranged below.
SORT CASES by PatientID(a) Dispensing_date(a).
EXECUTE.

*Rearrange the two rows per patient in ONE row per patient. In this dataset 69 patients remain.
SORT CASES BY PATIENTID dispensing_date.
CASESTOVARS
  /ID=PATIENTID
  /GROUPBY=VARIABLE.

*Save this file under another name, for now the rows of patients are aggregated.
SAVE OUTFILE=!path +'all_cascades_rearranged.sav'.

 *Now all the patients with the first episode meeting the inclusion criteria are selected, and the mean age, sex, time of onset for the ADR, and aSR can be calculated
*Calculation of the mean age over the whole population (in this database mean age 68 years with a standard deviation of 15).
USE ALL.
DESCRIPTIVES VARIABLES=age
  /STATISTICS=MEAN STDDEV MIN MAX.

*Calculation of the sex over the whole population (in this database 52.2% of patients is female).
USE ALL.
FREQUENCIES VARIABLES=Sex_code
  /ORDER=ANALYSIS.

*To indicate the time of onset of the second medication, calculate the mean exposure window for all positive values.
*An exposure window>0 indicates the sequence index medication followed by marker medication (in this database the mean exposure window was 123.6 days).
USE ALL.
COMPUTE filter_$=(exposure_window >= 0).
VARIABLE LABELS filter_$ 'exposure_window >= 0 (FILTER)'.
VALUE LABELS filter_$ 0 'Not Selected' 1 'Selected'.
FORMATS filter_$ (f1.0).
FILTER BY filter_$.
EXECUTE.
DESCRIPTIVES VARIABLES=exposure_window
  /STATISTICS=MEAN STDDEV MIN MAX.


*STEP 14 Calculating the aSR
*From this point, the aSR will be calculated. For an extensive explanation, see Appendix I ‘Calculations in PSSA’.
*Generate a new variable to count how many indexes there are followed by a marker medication (indexmarker) are vice versa (markerindex) in the complete dataset.
*During the aSR calculation several datasets are saved during the several steps to be able to return to a dataset or perform double checks.
USE ALL.
COUNT indexmarker=IndexORmarker.1(1).
VARIABLE LABELS indexmarker 'counting the number of patiens starting with an index followed by a marker'.
COUNT markerindex=IndexORmarker.1(2).
VARIABLE LABELS markerindex 'counting the number of patiens starting with a marker followed by an index'.
EXECUTE.

*Generate a new variable that counts the sum of patients with the sequence index followed by marker medication and vice versa.
CREATE sum_im=CSUM(indexmarker).
```

```
VARIABLE LABELS sum_im 'the sum of patiens starting with an index followed by a marker'.
CREATE sum_mi=CSUM(markerindex).
VARIABLE LABELS sum_mi 'the sum of patiens starting with a marker followed by an index'.

*The last row of variable sum_im contains the cumulative sum of the number of patients who have the sequence index
followed by a marker medication. The command below copies this cumulative sum value to all rows.
SORT CASES sum_im(d).
IF sum_im<lag(sum_im) sum_im=lag(sum_im).
EXECUTE.

* The last row of variable sum_mi contains the cumulative sum of the number of patients who have the sequence
marker followed by an index medication. The command below copies this cumulative sum value to all rows.
SORT CASES sum_mi(d).
IF sum_mi<lag(sum_mi) sum_mi=lag(sum_mi).
EXECUTE.

*The crude sequence ratio (cSR) could be calculated now.
*The cSR is 1.56 in this dataset.
COMPUTE cSR=sum_im/sum_mi.
VARIABLE LABELS cSR 'the crude sequence ratio for this dataset'.
EXECUTE.

*Save this dataset in the same path as defined in step 1. This dataset is needed later on.
SAVE OUTFILE=!path+'cSR.sav'.

*Give each row a number.
COMPUTE casenum=$casenum.
EXECUTE.

*Save this dataset as “indexmarker_markerindex” for the case numbering which is needed later on.
SAVE OUTFILE=!path+'indexmarker_markerindex.sav'.
DATASET NAME indexmarker_markerindex.
GET FILE=!path+'cSR.sav'.
DATASET CLOSE indexmarker_markerindex.

*The dates when a very first index medication was started needs to be extracted as Pa is calculated per day (see
Appendix 1). The command below extracts only the variable date_index and COMPUTEs on which dates a date_index
is dispensed.
SORT CASES BY date_index.
AGGREGATE
  /OUTFILE=!path+'date_index.sav'
  /PRESORTED
  /BREAK=date_index
  /number_index=N.

*Calculate the cumulative sum value for date_index to calculate the number of patient with an index during the study
period, and save the dataset as “date_index2”.
GET FILE=!path+'date_index.sav'.
COMPUTE sum_index=number_index.
VARIABLE LABELS number_index 'the number of patients that received an index medication at a certain date'.
EXECUTE.
IF lag(number_index)<9999 sum_index=sum_index+lag(sum_index).
VARIABLE LABELS sum_index 'the number of patients that received an index medication during the study period'.
EXECUTE.
SAVE OUTFILE=!path+'date_index2.sav'.

GET FILE=!path+'cSR.sav'.

* The dates when a very first marker medication was started needs to be extracted as Pa is calculated per day (see
Appendix 1). The command below extracts only the variable date_marker and COMPUTEs on which dates a
```

```
date_maker is dispensed.
SORT CASES BY date_marker.
AGGREGATE
/OUTFILE=!path+'date_marker.sav'
  /PRESORTED
  /BREAK=date_marker
  /number_marker=N.

*Calculate the cumulative sum value for date_marker of all the markers, i.e. how many patients received a marker medication at a certain date, and save the dataset as "date_marker".
GET FILE=!path+'date_marker.sav'.
COMPUTE sum_marker=number_marker.
VARIABLE LABELS number_marker 'the number of patients that received a marker medication at a certain date'.
EXECUTE.
IF lag(number_marker)<9999 sum_marker=sum_marker+lag(sum_marker).
VARIABLE LABELS sum_marker 'the number of patients that received a marker medication during the study period'.
EXECUTE.
SAVE OUTFILE=!path+'date_marker2.sav'.

*Match dataset date_index2 with only dates.
*The only dates dataset contains the data of the complete study period (here from 01/01/2015 until 31/12/2020). This is needed to match all dates between the study period with the number of times a first index was dispensed.
MATCH FILES
    /FILE=!path+'from 3.sav'
    /FILE=!path+'date_index2.sav'
    /BY date_index.
EXECUTE.
SAVE OUTFILE=!path+'date_and_index.sav'.

*Match dataset ”date_marker2” with dataset "date_and_index".
MATCH FILES
    /FILE=!path+'date_and_index.sav'
    /FILE=!path+'date_marker2.sav'
/BY date_marker.
EXECUTE.
SAVE OUTFILE=!path+'date_and_index_and_marker.sav'.

*Add a 0 where the values are missing (i.e. there has been no dispensing of the index nor the marker medication on that specIFic date).
RECODE number_index number_marker (SYSMIS=0).
EXECUTE.

*Clean up the dataset as only one date variable is needed.
DELETE VARIABLES date_marker.
RENAME VARIABLES (date_index=date).

*Calculate over the study period each addition of a new index (or marker) medication per day over time. Note that every time an index medication is prescribed the sum_index also adds up (and the same for the marker).
FORMATS sum_index sum_marker (f4.0).
IF sysmis(sum_index) sum_index=lag(sum_index).
IF sysmis(sum_marker) sum_marker=lag(sum_marker).
EXECUTE.
IF sysmis(sum_index) sum_index=0.
IF sysmis(sum_marker) sum_marker=0.
EXECUTE.

*The exposure window in this study was 12 months. Therefore, for the first part of the denominator of the Pa formula, for each date 12 months before and 12 months after that date are calculated.
*Note that the years 2021 (12 month after the date) and 2014 (12 months before the date) are added.
COMPUTE date2=date+(365*24*60*60).
```

VARIABLE LABELS date2 '12 months after the date'.
COMPUTE date3=date-(365*24*60*60).
VARIABLE LABELS date3 '12 months before the date'.
EXECUTE.
FORMATS date2 date3(edate10).
SAVE OUTFILE=!path+'tmp1.sav'.

*Save the dataset as a temporary dataset with only the date, and the sums of the index and marker over the study period. .
SAVE OUTFILE=!path+'tmp2.sav'
    /keep date sum_index sum_marker.

*Looking a year ahead of time: rename the variables that are needed later to match files.
GET FILE=!path+'tmp2.sav'.
RENAME VARIABLES (date=date2) (sum_index=sum_index2) (sum_marker=sum_marker2).
VARIABLE LABELS sum_index2 'the number of patients that received an index medication one year ahead of time'.
VARIABLE LABELS sum_marker2 'the number of patients that received a marker medication one year ahead of time'.
SAVE OUTFILE=!path+'tmp3.sav'.

*Looking a year back in time: rename the variables that are needed later to match files.
GET FILE=!path+'tmp2.sav'.
RENAME VARIABLES (date=date3) (sum_index=sum_index3) (sum_marker=sum_marker3).
VARIABLE LABELS sum_index3 'the number of patients that received an index medication looking one year back in time'.
VARIABLE LABELS sum_marker3 'the number of patients that received a marker medication looking one year back in time'.

SAVE OUTFILE=!path+'tmp4.sav'.

*Match the correct sums of index and marker with the correct date. Thus, each row contains the sum of indexes and markers on that specific date and the sum of indexes and markers exactly one year ahead of that date.
MATCH FILES
    /FILE=!path+'tmp1.sav'
    /FILE=!path+'tmp3.sav'
    /BY date2.
EXECUTE.

*Select only the study period dates, since the year 2015 in this syntax was used for the washout window. (note that the year 2014 is removed, which was added by looking one year back in time).
SELECT IF date2>=date.dmy(1,1,2016).
EXECUTE.
SAVE OUTFILE=!path+'tmp5.sav'.

*Match the year after (tmp 5) with the year before (tmp4) with each other. Thus, now, to the rows is added the sum of dispensings exactly one year before.
MATCH FILES
    /FILE=!path+'tmp5.sav'
    /FILE=!path+'tmp4.sav'
    /BY date3.
EXECUTE.

*When looking one year ahead in time for the last study day (i.e. 31-12-2020 in this study) the last sum of marker medication needs to be copied for all cells from 1-1-2021.
SORT CASES by date2 (a).
IF date2>=date.dmy(1,1,2021) sum_marker2=lag(sum_marker2).
EXECUTE.

*When looking one year ahead in time from the start of the studyperiod (i.e. 1-1-2016 in this study as the year 2015 is excluded for the washout window): COMPUTE the number of markers medication patients are receiving.
SORT CASES by date (d).

```
IF date>=date.dmy(1,1,2016) markers_year_after=lag(sum_marker2)-sum_marker.
VARIABLE LABELS markers_year_after 'the number of patients receiving a marker medication looking one year ahead
from the observed date'.
EXECUTE.

*The same as above is done for looking one year back in time.
SORT CASES by date (a).
COMPUTE markers_year_before=lag(sum_marker)-lag(sum_marker3).
VARIABLE LABELS markers_year_before 'the number of patients receiving a marker medication looking one year back
in time from the observed date'.
EXECUTE.

*Calculate the numerator for one observed date (see formula Pa in appendix 1).
COMPUTE numerator=number_index*markers_year_after.
EXECUTE.

*Calculate the denominator for one observed date (see formula Pa in appendix 1).
COMPUTE denominator=number_index*(markers_year_after+markers_year_before).
EXECUTE.

*Calculate the sum of the numerator and denominator.
CREATE sum_numerator=CSUM(numerator).
CREATE sum_denominator=CSUM(denominator).

*Only select the last row, because this contains the summed-up value and to calculate the aSR only one row is needed.
SELECT IF date=date.dmy(31,12,2020).
EXECUTE.

*Put a case number in this row, so it can be matched.
COMPUTE casenum=$casenum.
EXECUTE.
SAVE OUTFILE=!path+'tmp6.sav'.

*Match the two files based on the case number.
MATCH FILES
  /FILE=!path+'tmp6.sav'
  /TABLE=!path+'indexmarker_markerindex.sav'
  /BY casenum.
EXECUTE.

*Calculate the variables to determine the aSR (see Appendix I where the formula is explained). Note that the crude
Sequence Ratio is calculated again here, as an additional check.
COMPUTE pa=sum_numerator/sum_denominator.
COMPUTE null_effect_SR=pa/(1-pa).
COMPUTE crude_SR=sum_im/sum_mi.
COMPUTE adjusted_SR=crude_SR/null_effect_SR.
COMPUTE low_CI95=adjusted_SR-1.96*(sqrt(1/sum_im+1/sum_mi)).
COMPUTE high_CI95=adjusted_SR+1.96*(sqrt(1/sum_im+1/sum_mi)).
EXECUTE.

*The calculated values in this dataset are: null_effect_sr=1.12, the adjusted_SR=1.39, the low_CI95=0.91 and the
high_CI95=1.87.
*Save the dataset as “result”.
SAVE OUTFILE=!path+'result.sav'.
GET FILE=!path+'result.sav'.

*Delete all the temporary datasets.
ERASE FILE=!path+'all_cascades_rearranged.sav'.
ERASE FILE=!path+'all_cascades.sav'.
ERASE FILE=!path+'all_episodes.sav'.
```

```
ERASE FILE=!path+'episodes1.sav'.
ERASE FILE=!path+'episodes2.sav'.
ERASE FILE=!path+'episodes3.sav'.
ERASE FILE=!path+'episodes4.sav'.
ERASE FILE=!path+'motherfile.sav'.
ERASE FILE=!path+'cSR.sav'.
ERASE FILE=!path+'tmp1.sav'.
ERASE FILE=!path+'tmp2.sav'.
ERASE FILE=!path+'tmp3.sav'.
ERASE FILE=!path+'tmp4.sav'.
ERASE FILE=!path+'tmp5.sav'.
ERASE FILE=!path+'tmp6.sav'.
ERASE FILE=!path+'indexmarker_markerindex.sav'.
ERASE FILE=!path+'date_and_index_and_marker.sav'.
ERASE FILE=!path+'date_and_index.sav'.
ERASE FILE=!path+'date_marker.sav'.
ERASE FILE=!path+'date_index.sav'.
ERASE FILE=!path+'date_marker2.sav'.
ERASE FILE=!path+'date_index2.sav'.
```
